# Insomnia Disorder in Adult Attention-Deficit/Hyperactivity Disorder Patients: Clinical, Comorbidity, and Treatment Correlates

**DOI:** 10.3389/fpsyt.2021.663889

**Published:** 2021-05-26

**Authors:** Christian Fadeuilhe, Constanza Daigre, Vanesa Richarte, Lara Grau-López, Raul F. Palma-Álvarez, Montse Corrales, Josep A. Ramos-Quiroga

**Affiliations:** ^1^Department of Psychiatry, Hospital Universitari Vall d'Hebron, Barcelona, Spain; ^2^Biomedical Network Research Centre on Mental Health (CIBERSAM), Barcelona, Spain; ^3^Group of Psychiatry, Mental Health and Addiction, Vall d'Hebron Research Institute, Barcelona, Spain; ^4^Department of Psychiatry and Forensic Medicine, Universitat Autònoma de Barcelona, Barcelona, Spain

**Keywords:** attention deficit and hyperactivity disorder, insomnia disorder, adult ADHD, sleep disorder, severity, comorbidity

## Abstract

**Introduction:** Several investigations have been performed on insomnia symptoms in adult attention-deficit/hyperactivity disorder (ADHD). However, the relationship between insomnia disorder and adult ADHD has been neglected in research. The main objective of the current study is to analyze the differences between adult ADHD patients with and without insomnia disorder, in terms of ADHD clinical severity, medical and psychiatric comorbidity, psychopharmacological treatment, and quality of life.

**Material and Methods:** Two hundred and fifty-two adult patients with ADHD (mean age 37.60 ± 13.22 years; ADHD presentations—combined: 56.7%, inattentive: 39.7%, hyperactive/impulsive: 3.6%) were evaluated with an exhaustive clinical and psychological evaluation protocol including semistructured interviews (for comorbidities and ADHD assessment) and symptom rating scales for ADHD. The diagnosis of ADHD and insomnia disorder was made according to DSM-5 criteria. Furthermore, the Pittsburgh Sleep Quality Index, Insomnia Severity Index, and Epworth Sleepiness Scale were administered.

**Results:** Insomnia disorder was found in 44.4% of adult ADHD patients and was more common in combined presentation (64.3%) and in patients with more ADHD severity. Comorbidities (both medical and psychiatric), especially mood disorders (42%), anxiety disorder (26.8%), personality disorder (39.3%), and any substance use disorder (11.6%), were associated with a higher insomnia disorder prevalence. ADHD stimulant treatment was related to lower insomnia disorder compared to patients without medication, as well as ADHD stable treatment. Additionally, worse health-related quality of life was associated with insomnia disorder.

**Conclusion:** Insomnia disorder is highly prevalent in adult ADHD and is related to higher ADHD severity and more psychiatric and medical comorbidities. Some stimulants and stable pharmacological ADHD treatment are associated with better outcomes of insomnia disorder.

## Introduction

Attention-deficit/hyperactivity disorder (ADHD) is a neurodevelopmental disorder characterized by the presence of persistent symptoms of inattention and/or hyperactive–impulsive behavior (1, 2). The Diagnostic and Statistical Manual of Mental Disorders 5th edition (DSM-5) (1) requires for adult ADHD diagnoses five or more symptoms of inattention and/or of hyperactivity/impulsivity. These symptoms should be present before the age of 12, have lasted 6 months, and interfere with daily life activities. ADHD frequently persists into adulthood up to 40–60%, with the prevalence of ADHD in adults ranging between 2 and 3% (3). Adult ADHD has been related to several psychiatric comorbidities (note that comorbidity refers to any co-occurring disorder associated to ADHD), including anxiety disorders, mood disorders, substance use disorders (SUD), behavior disorders, and sleep disorders (2).

Insomnia disorder is one of the most frequent health problems in the adult general population, with an estimated prevalence between 6 and 15% (4, 5). However, less than half who suffer from insomnia disorder receive any type of treatment (6, 7). According to the DSM-5 (1), the insomnia disorder diagnosis is reached if there is a predominant dissatisfaction in the quantity or quality of sleep for at least three nights a week for at least 3 months in initiating or maintaining sleep or early morning awakening, causing significant discomfort or clinical impairment in any area of the person's functioning.

In children, several researchers have reported a significant association between ADHD and sleep disturbances, using objective (actigraphy and polysomnography) and subjective studies (8–10). In adults, the relationship between ADHD and sleep disorders has been scarcely studied and several questions and controversies are unresolved (8).

The prevalence of insomnia symptoms in this population has a wide range between 43 and 85% (11–14), being more prevalent in women (15). In this vein, there are discrepancies regarding the relationship between insomnia and some variables associated with ADHD (11, 16). Considering the different presentations of ADHD, some investigations did not report differences (17), whereas others report poorer quality of sleep in inattentive presentation than in the combined presentation (13). However, other studies indicate that sleep problems are more frequently associated with combined or hyperactive/impulsive presentation (11, 18). Besides, ADHD severity may be associated with worse insomnia presentation (19). The severity of insomnia and the consequent presence of daytime sleepiness has been related to a greater number of ADHD symptoms (20).

Insomnia symptoms and several psychiatric comorbidities have a bidirectional relationship. Mental comorbidities increase the risk of insomnia symptoms (21), and insomnia symptoms can also increase psychiatric comorbidity severity (19). In adult ADHD patients, depressive symptoms, substance use disorder, and family history of mood disorders have been related to more insomnia symptoms (19, 21). In any case, few studies have been conducted on this issue and their results are heterogeneous among adult ADHD (22).

The relationship between ADHD treatment and insomnia is not well-established. Van Veen et al. reported that about 80% of untreated ADHD patients had sleep-onset insomnia symptoms (23). On the other hand, atomoxetine and psychostimulants can induce insomnia symptoms as an adverse effect (24, 25). However, after 2 months, this adverse effect seems to be mitigated (26, 27), and some patients report improvement of insomnia symptoms with methylphenidate (11).

Most of the studies have focused on insomnia symptoms instead of insomnia disorder, with insomnia disorder in adult ADHD patients being frequently neglected in research. Hence, the main objective of the current study is to analyze the differences between adult ADHD patients with and without insomnia disorder, in terms of ADHD clinical severity, medical and psychiatric comorbidity, psychopharmacological treatment, and quality of life. We hypothesize that insomnia disorder would be associated with greater clinical ADHD severity and psychiatric comorbidities, an unstable ADHD treatment, and a poorer health-related quality of life.

Furthermore, we intend to analyze the differences between patients with insomnia disorders and those with insomnia symptoms. It is hypothesized that insomnia disorder would be associated with greater clinical ADHD severity and psychiatric comorbidities.

## Materials and Methods

### Study Design and Patients

This cross-sectional study was conducted at the Adult ADHD Program of the Hospital Universitari Vall d'Hebron in Barcelona (Spain). Participants referred for the first time to the ADHD Program were recruited consecutively between April 2019 and February 2020 whether ADHD diagnosis was confirmed. This study was approved by the Clinical Research Ethics Committee of the Hospital Universitari Vall d'Hebron. All the participants voluntarily agreed to get involved in the study and did not receive any financial compensation.

The participants fulfilled the following inclusion criteria: were over 18 years, met ADHD criteria according to DSM-5, accepted to participate, and signed the informed consent. Those patients diagnosed with a sleep disorder other than insomnia disorder (e.g., circadian rhythm disorder), intellectual disability (intelligence quotient < 70), or any medical condition that could explain the symptoms of ADHD (such as hearing and vision impairments, epilepsy, traumatic brain injury, or thyroid disease) were excluded.

### Instruments and Variables

#### ADHD Diagnosis

The *Conners' Adult ADHD Diagnostic Interview for DSM-IV part I* (CAADID-I) (28) is a semistructured interview that collects information on ADHD from childhood to adulthood. This instrument has good sensitivity (98.86%) and specificity (67.68%). The diagnostic precision was 91.46% and the *kappa* index concordance was 0.88 (28).

The *Diagnostic Interview for ADHD in Adults* (DIVA 2.0) (29) consists of a structured interview for ADHD that evaluates in adults each of the 18 symptom criteria for ADHD, in childhood and adulthood. This has good sensitivity (90.0%) and specificity (72.9%) (30).

The *Conners' Adult ADHD Rating Scales—Long Version* (CAARS) is a 66-item self-reported instrument designed to assess ADHD in adults and has high degrees of sensitivity and specificity, with an overall diagnostic efficiency rate of 85% (31).

All these instruments were used to assess and confirm ADHD diagnosis. They have been validated in a Spanish population, have good psychometric properties in ADHD diagnosis (28, 29, 32), and were used based on DSM-5 criteria.

#### ADHD Severity

The clinical severity of ADHD was evaluated by using the *ADHD Rating Scale* (ADHD-RS). It is a self-administered 18-item validated scale that provides a consistent way for clinicians to diagnose ADHD according to the DSM-5 criteria, with high sensitivity (81.9%) and specificity (74.7%) and a kappa coefficient of 0.78 (33). Also, the *Clinical Global Impression—Severity Scale* (CGI-S) was used. It is a reference scale in the evaluation of the disorders severity and has also shown good psychometric properties (34).

#### Psychiatric Comorbidities and Psychological Characteristics

The *Structured Clinical Interviews for Axis I* (SCID-I) *and Axis-II* (SCID-II) (35) were used to evaluate patients' psychiatric comorbidities other than ADHD and insomnia disorder. SCID is considered the gold standard for all psychiatric research to increase reliability of diagnostic assessment and minimize clinical judgment that could lead to unreliable diagnoses (36).

The *Beck-II Depression Inventory II* (BDI-II) was administered for measuring depressive symptomatology (37). This reference instrument in depression research has good sensitivity (89.6%) and specificity (86.5%) (38).

The *State-Trait Anxiety Inventory* (STAI) was used to measure anxiety (both trait and state) (39). It is one of the most widely used scales to measure anxiety both in the clinic and in research (Cronbach's alpha = 0.91 for anxiety state and 0.89 for anxiety trait) (40).

The *Barratt Impulsiveness Scale* (BIS-11) was used for measuring trait impulsivity as this instrument provides a total score and three subscale scores (cognitive impulsivity, motor impulsivity, and unplanned impulsivity), and therefore, this scale measures impulsivity as a multidimensional construct with good reliability (Cronbach's alpha = 0.83) (41).

#### Insomnia and Other Sleep Disorders

Three instruments were used to evaluate insomnia, sleep, and related disorders. The first instrument was the *Insomnia Severity Index* (ISI) which evaluates insomnia severity in the last month and includes several sleep dimensions: severity of sleep onset, sleep maintenance, early morning awakening problems, sleep dissatisfaction, interference of sleep difficulties with daytime functioning, noticeability of sleep problems by others, and distress caused by the sleep difficulties (42). The ISI sensitivity was 82.4% and its specificity was 82.1% (43). The second instrument was the *Pittsburgh Sleep Quality Index* (PSQI). This is a 19-item self-reported instrument that assesses sleep quality and its disturbances during the last month (44). The PSQI evaluates seven components: sleep quality, sleep latency, sleep duration, habitual sleep efficiency, sleep disturbance, use of sleeping medication, and daytime dysfunction. It is interpreted as the higher the score, the worse the subjective quality of sleep (45). The reported psychometric values are 89.6% for sensitivity and 86.5% for specificity (44). Finally, the third instrument used was the *Epworth Sleepiness Scale* (ESS) which is a self-reported questionnaire with eight items that allows measuring the subject's overall level of daytime sleepiness (46) with 70% sensitivity and 55.6% specificity (47). These instruments are validated and have good psychometric features for sleep assessment in clinical research.

#### Health-Related Quality of Life Evaluation

The short-form questionnaire SF-36 was used for assessing the health-related quality of life (HRQoL) (48). This instrument has eight domains (physical functioning, role limitations due to physical problems, bodily pain, general health perceptions, vitality, social functioning, role limitations due to emotional problems, and mental health) that can be aggregated into two summary measures: physical component summary (PCS) and mental component summary (MCS). With a Cronbach's alpha > 0.85 (49), this instrument is one of the most accurate for measuring HRQoL.

### Procedure

The Adult ADHD Program of the Hospital Universitari Vall d'Hebron is a multidisciplinary and comprehensive program that evaluates and treats referred patients from primary care centers, community mental health centers, and addiction treatment units. Once referred to the ADHD Program, a complete assessment of the patient is carried out to establish the diagnosis and the treatment.

For the current study, the evaluation process consisted of five visits conducted by trained staff specialized in ADHD and sleep disorders (psychiatrists and psychologists). At the first visit, the psychiatrist obtains a complete medical history, including a clinical approach for ADHD and insomnia disorder. Besides clinical assessment, a standardized evaluation with validated instruments for ADHD (CAADID-I, DIVA 2.0, CGI-S) and insomnia disorder (ISI, ESS, and PSQI) was conducted during the first visit. At the next three visits, the psychologist carried out the CAARS, ADHD-RS, STAI, BDI, SF-36, SCID-I, and SCID-II interviews. Finally, at the fifth visit, the psychiatrist responsible for the patients reviewed all the information and communicated the final diagnosis to the patient at the end of the study. The diagnosis of insomnia disorder and ADHD was made by the psychiatrist according to DSM-5 criteria. Whether the diagnosis of ADHD was confirmed, the patient was offered to perform a psychiatric and psychological treatment and follow-up in the program (the treatment offered adheres to the clinical guidelines for ADHD and insomnia disorder).

The Wechsler Adult Intelligence Scale-IV (WAIS-IV) was used to assess patients' intelligence quotient (IQ).

### Statistical Analysis

Bivariate and multivariate analyses were executed using SPSS version 20 for Windows. First, a descriptive analysis of all variables as percentages, means, and standard deviations was conducted. To compare ADHD patients with and without insomnia disorder, chi-square test was used for nominal variables and Student's *t*-test and ANOVA were used for quantitative variables. Bonferroni correction for multiple testing was used in order to minimize type I error. Finally, a logistic regression analysis was conducted using the variables that remained statistical significance Bonferroni correction. In order to avoid collinearity, variables related to sleep were not included in the multivariate analyses. The dependent variable was insomnia disorder diagnosis (0 = no insomnia disorder and 1 = insomnia disorder). A conditional entrance method was used to select variables in the model. All statistical hypotheses were two-sided and a value of *p* < 0.05 was considered statistically significant.

## Results

### Sample Recruitment and Sample Features

During the recruitment period, 252 patients (58% males; mean age 37.6 ± 13.2 years old) met the inclusion criteria and accepted to participate in the study. In order of frequency, the combined presentation of ADHD was the most common (56.7%), followed by the inattentive presentation (39.7%) and hyperactive/impulsive presentation (3.6%). Regarding insomnia, 44.4% of patients met the criteria for insomnia disorder and 63.9% of the total sample participants had insomnia symptoms.

### Results in Relation to the Presence of Insomnia Disorder

Sociodemographic, clinical, and psychometric variables are presented in Table 1 according to the presence of insomnia disorder. Regarding the bivariate analysis, there were no differences in sociodemographic features except for employment characteristics (ADHD patients with insomnia disorder were more unemployed). No significant differences were found on IQ between patients with insomnia disorder (101.08 ± 11.91; *T*: 1.467; *p* = 0.144) and those without that disorder (103.42 ± 13.10) and neither also when family psychiatric records were compared. Any medical record and high body index mass (BMI) were related to a higher prevalence of insomnia disorder. Regarding specific medical comorbidities, due to the small sample size, some medical diseases could not be statistically analyzed. However, it was observed that the group with insomnia disorder had a higher prevalence of pain syndrome compared with the group without insomnia disorder (18.9 vs. 13.2%) but a lower prevalence rate regarding diabetes mellitus (1.9 vs. 7.9%) and arterial hypertension (3.8 vs. 18.4%). No differences were found regarding other comorbidities, including cardiac, endocrine, gastrointestinal, respiratory, and neurological diseases. Similarly, several pharmacological treatments for those medical diseases were not analyzed because of small size. In any case, non-opioid analgesics were more used in patients with insomnia disorder (29.5 vs. 19.4%), while a fewer use of antihypertensive (4.5 vs. 22.6%) and oral antidiabetic medications (2.3 vs. 9.7%) was observed. No differences were found for other medication categories for medical conditions (e.g., endocrinological, neurological, cardiologic, and gastrointestinal medications) and psychiatric disorders (e.g., antidepressants, anxiolytics, anticonvulsants, and antipsychotics).

**Table 1:** Sociodemographic and clinical characteristics according to insomnia disorder.

	**Total** **% (*n*) or mean ± SD**	**Insomnia disorder** **% or mean** **±** **SD**		***X*^**2**^ or *t***	***p***
		**Yes (44.4%)**	**No (55.6%)**		
**Sociodemographic features**					
Sex (males)	58.7 (148)	58	59.3	0.4	0.841
Age	37.60 ± 13.22	38.49 ± 12.25	36.86 ± 13.95	0.967	0.334
Nationality (Spanish)	90.9 (229)	87.5	93.6	2.765	0.096
Marital status (single)	32.1 (81)	31.3	32.9	0.074	0.786
Level of studies (university studies)	56.3 (142)	50	61.4	3.304	0.069
Employment (employment)	59.5 (150)	52.7	65	6.098	0.047
**Clinical characteristics**					
Family psychiatric records	51.6 (130)	58	46.4	3.357	0.067
BMI	25.25 ± 4.41	26.24 ± 5.01	24.46 ± 3.70	3.126	0.020
Any medical records	36.5 (92)	47.3	27.9	10.170	0.001^*^
Previous psychiatric comorbidities	63.1 (159)	76.8	52.1	16.227	< 0.0001^*^
Mood disorders	44.0 (111)	53.6	36.4	7.419	0.006
Depressive disorders	27.0 (68)	32.1	22.9	2.723	0.099
Adaptation disorders	23.0 (58)	30.4	17.1	6.132	0.013
Anxiety disorders	32.5 (82)	45.5	22.1	15.511	< 0.0001^*^
Bipolar disorders	0.8 (2)	0.9	0.7	0.025	0.874
Substance use disorders	23.8 (60)	34.8	15	13.476	< 0.0001^*^
Alcohol use disorder	12.7 (32)	18.8	7.9	6.660	0.010
Cannabis use disorder	17.9 (45)	24.1	12.9	5.369	0.021
Cocaine use disorder	10.3 (26)	15.2	6.4	5.149	0.023
Any current psychiatric comorbidities	49.2 (124)	69.6	32.9	33.688	< 0.0001^*^
Current psychiatric comorbidities (without PD)	46.0 (116)	66.1	30	32.589	< 0.0001^*^
Any mood disorders	26.2 (66)	42	13.6	25.948	< 0.0001^*^
Depressive disorders	8.7 (22)	15.2	205	10.521	0.001
Adaptation disorders	17.5 (44)	26.8	10	12.165	< 0.0001
Anxiety disorders	17.9 (45)	26.8	10.7	10.957	0.001
Bipolar disorders	0.8 (2)	0.9	0.7	0.250	0.874
Autism disorders	4.0 (10)	3.6	4.3	0.083	0.773
Any substance use disorders	7.5 (19)	11.6	4.3	4.784	0.029
Alcohol use disorders	4 (10)	6.2	2.1	2.754	0.097
Cannabis use disorders	4.4 (11)	6.2	2.9	1.716	0.190
Cocaine use disorders	2 (5)	3.6	0.7	2.612	0.106
Any personality disorders	27.4 (69)	39.3	17.9	14.369	< 0.0001
Cluster A personality disorder	0.8 (2)	1.8	0	2.52	0.112
Cluster B personality disorder	10.7 (27)	16.1	6.4	6.048	0.014
Cluster C personality disorder	12.7 (32)	16.1	10	2.069	0.150
Personality disorder not specified	3.2 (8)	3.6	2.9	0.103	0.748
**Psychometric features**					
Depressive symptoms (BDI)	11.44 ± 10.27	15.81 ± 10.74	7.95 ± 8.41	6.344	< 0.0001^*^
Anxiety trait (STAI)	28.73 ± 12.81	34.84 ± 11.25	23.84 ± 11.87	7.485	< 0.0001^*^
Anxiety state (STAI)	25.86 ± 13.85	32.14 ± 13.73	20.84 ± 11.76	7.038	< 0.0001^*^
Impulsivity (BIS-11)	66.45 ± 16.08	70.93 ± 13.82	62.86 ± 16.89	4.078	< 0.0001^*^
Physical component summary of HRQoL (SF-36)	49.79 ± 8.47	47.15 ± 9.05	51.91 ± 7.35	4.509	< 0.0001^*^
Mental component summary of HRQoL (SF-36)	41.86 ± 11.32	34.26 ± 9.07	47.94 ± 9.04	11.916	< 0.0001^*^

*PD, personality disorder*.

* *The result is statistically significant after Bonferroni correction*.

ADHD patients with any psychiatric comorbidity are more likely to present insomnia disorder, especially those who currently have mood disorders, anxiety disorder, any SUD, and any personality disorder (mainly cluster B personality disorders). Congruently, ADHD patients with insomnia disorder had higher BDI-II, STAI, and BIS-11 scores. Finally, a worse HRQoL (both physical and mental score) was observed in ADHD patients with insomnia disorder (Table 1).

Regarding ADHD variables and insomnia disorder, it was observed that insomnia disorder was most frequently presented in patients with the combined presentation of ADHD or with higher severity of ADHD (according to CGI, CAARS, and ADHD-RS). Patients who were in any pharmacological treatment had less prevalence of insomnia disorder (specifically those receiving methylphenidate). Longer periods of stable treatment were associated with lower rates of insomnia disorder (Table 2).

**Table 2:** ADHD variables related to insomnia disorder.

**Variables**	**Total** **% (*n*) or mean ± SD**	**Insomnia disorder** **% or mean** **±** **SD**		***X*^**2**^ or *t***	***p***
		**Yes (44.4%)**	**No (55.6%)**		
**ADHD presentation**					
Inattentive	39.7 (100)	31.2	46.4	6.082	0.048
Hyperactive/impulsive	3.6 (9)	4.5	2.9		
Combined	56.7 (143)	64.3	50.7		
**Clinical global impression—severity scale**					
Borderline mentally ill (2)	6.0 (15)	0	10.7	101.134	<0.0001^*^
Mildly ill (3)	32.5 (82)	10.7	50		
Moderately ill (4)	25.0 (63)	20.5	28.6		
Markedly ill (5)	27.8 (70)	50.9	9.3		
Severely ill (6)	8.7 (22)	17.9	1.4		
Extremely ill (7)	0 (0)	0	0		
**ADHD evaluation**					
DIVA 2.0 (A)	7.29 ± 1.20	7.53 ± 1.27	7.11 ± 1.11	2.792	0.006
DIVA 2.0 (H/I)	5.05 ± 2.77	5.68 ± 2.81	4.55 ± 2.64	3.28	0.001^*^
CAARS (direct score)	20.87 ± 6.84	22.87 ± 5.85	19.28 ± 7.17	4.376	<0.0001^*^
ADHD-RS	26.02 ± 10.75	31.46 ± 9.69	21.67 ± 9.52	8.039	<0.0001^*^
**Pharmacological treatment**					
Any pharmacological treatment	59.5 (150)	51.8	65.7	5.01	0.025
Methylphenidate	34.1 (86)	25.9	40.7	6.08	0.014
Lisdexamfetamine	19.4 (49)	22.3	17.1	1.065	0.302
Atomoxetine	6.3 (16)	4.5	7.9	1.205	0.272
Guanfacine	1.6 (4)	0	2.9	3.252	0.071
Months in stable treatment	12.32 ± 15.89	4.97 ± 7.89	18.19 ± 18.11	7.767	<0.0001^*^

* *The result is statistically significant after Bonferroni correction*.

*DIVA (A), Diagnostic Interview for ADHD in Adults criteria for attention deficit; DIVA (H/I), Diagnostic Interview for ADHD in Adults criteria for hyperactivity-impulsivity*.

Regarding sleep assessment, patients with insomnia disorder presented higher scores on the ISI scale and the PSQI. When the PSQI subscales were analyzed, statistically significant results were reported. Likewise, patients with insomnia disorder had greater daytime sleepiness reported on the ESS. Finally, both the prescription of hypnotic medications and their number were higher in patients with insomnia disorder. No differences were found among the different groups of hypnotic medications and in the concomitant prescription of other psychotropic drugs (Table 3).

**Table 3:** Sleep variables related to insomnia disorder.

**Variables**	**Total** **% (*n*) or mean ± SD**	**Insomnia disorder** **% or mean** **±** **SD**		***X*^**2**^ or *t***	***p***
		**Yes (44%)**	**No (55.6%)**		
Screen use	71.4 (180)	87.5	58.6	25.515	<0.0001^*^
Total sleep time (h)	6.63 ± 1.32	5.82 ± 1.13	7.28 ± 1.09	10.42	<0.0001^*^
Insomnia severity index	8.96 ± 8.72	18.08 ± 4.07	1.67 ± 1.85	39.504	<0.0001^*^
Epworth sleepiness scale	5.46 ± 4.63	8.83 ± 3.30	2.76 ± 3.68	13.632	<0.0001^*^
Pittsburgh sleep quality index global score	6.98 ± 5.40	12.12 ± 3.33	2.87 ± 2.38	25.659	<0.0001^*^
**Sleep treatment variables**					
Use of sleep medication	15.9 (40)	25.9	7.9	15.157	<0.0001^*^
**Sleep medication**					
Benzodiazepines	7.9 (20)	15.2	2.1	14.471	<0.0001^*^
Antidepressants	4.4 (11)	6.2	2.9	1.716	0.190
Antipsychotics	3.2 (8)	3.6	2.9	0.103	0.748
Anticonvulsants	3.6 (9)	5.4	2.1	1.867	0.172
Melatonin	2.0 (5)	3.6	0.7	2.612	0.106
Psychiatric comorbidity medication without hypnotic effect	24.2 (61)	29.5	20	4.438	0.109
Number of baseline medication	0.22 ± 0.58	0.36 ± 0.70	0.11 ± 0.43	3.389	0.001^*^
Number of medications after first assessment	0.43 ± 0.85	0.83 ± 1.06	0.11 ± 0.41	7.385	<0.0001^*^

* *The result is statistically significant after Bonferroni correction*.

When the different insomnia phenotypes were analyzed, sleep-onset insomnia was the most frequent (32.9%), followed by sleep maintenance insomnia (31.0%), mixed insomnia (25.4%), and early morning awakening (11.5%). These phenotypes were compared based on sociodemographic, ADHD, and psychiatric comorbidity characteristics. No statistically significant differences were found, except for age. Younger age was associated with sleep-onset insomnia (29.32 ± 9.50; *F* = 8.426; *p* ≤ 0.001), while in older patients, maintenance insomnia (40.78 ± 12.80), mixed insomnia (41.70 ± 11.43), and early awakening insomnia were the predominant phenotypes (43.50 ± 6.36).

Insomnia disorder and insomnia symptoms groups were characterized and compared in terms of contribution to cast more insight on the role of specific factors influencing the transition between them. Compared with the insomnia symptoms group, it was observed that patients with insomnia disorder presented more current psychiatric comorbidities; higher depression, anxiety, and impulsivity psychometric scores; and a higher severity of ADHD according to ADHD-RS. The logistic regression showed that the months of stable ADHD treatment and the mental quality of life were greater among the patients with insomnia symptoms than among the patients with insomnia disorder and were independently associated (*X*^2^ = 66.932; *p* ≤ 0.0001, Nagelkerke *R*^2^ = 0.481; constant value 1.129). On the other hand, no differences were found comparing personality disorders and SUD variables. Likewise, no differences were reported considering the different ADHD medications (Table 4).

**Table 4:** Sociodemographic and clinical features of ADHD patients according to insomnia disorder or insomnia symptoms.

	**Total** **% (*n*) or mean ± SD**	**Insomnia disorder** **(*n* = 112)**	**Insomnia symptoms** **(*n* = 49)**	***X*^**2**^ or *t***	***p***
	**(*n* = 161)**	**% or mean** **±** **SD**			
**Sociodemographic features**					
Sex (males)	59.6 (96)	58	63.3	0.387	0.602
Age	38.51 ± 12.70	38.49 ± 12.25	35.55 ± 13.79	0.27	0.978
**Clinical characteristics**					
BMI	25.69 ± 4.69	26.24 ± 5.01	24.43 ± 3.59	2.581	0.011
Any medical records	43.5 (70)	47.3	34.7	2.212	0.168
Any current psychiatric comorbidities	60.9 (98)	69.6	40.8	11.892	0.001^*^
Any mood disorders	35.4 (57)	42.0	20.4	6.926	0.012
Anxiety disorders	23.0 (37)	26.8	14.3	3.009	0.083
Bipolar disorders	0.6 (1)	0.9	0		NA
Autism disorders	4.3 (7)	3.6	6.1	0.533	0.465
Any substance use disorders	9.3 (15)	11.6	4.1	2.285	0.154
Any personality disorders	34.2 (55)	39.3	22.4	4.296	0.047
**Psychometric features**					
Depressive symptoms (BDI)	13.82 ± 10.71	15.81 ± 10.74	9.27 ± 9.20	3.710	<0.0001^*^
Anxiety trait (STAI)	34.46 ± 11.98	34.84 ± 11.25	27.02 ± 11.92	3.985	<0.0001^*^
Anxiety state (STAI)	29.50 ± 13.68	32.14 ± 13.73	23.45 ± 11.58	3.869	<0.0001^*^
Impulsivity (BIS-11)	68.61 ± 14.77	70.93 ± 13.82	63.31 ± 15.62	3.093	0.002^*^
Physical component summary of HRQoL (SF-36)	48.26 ± 9.14	47.15 ± 9.05	50.80 ± 8.92	2.368	0.019
Mental component summary of HRQoL (SF-36)	38.02 ± 10.74	34.26 ± 9.07	46.62 ± 9.27	7.900	<0.0001^**^
**ADHD variables**					
ADHD presentation					
Inattentive	35.4 (57)	31.2	44.9	2.800	0.247
Hyperactive/impulsive	4.3 (7)	4.5	4.1		
Combined	60.2 (97)	64.3	51.0		
**Clinical global impression—severity scale**					
Borderline mentally ill (2)	3.1 (5)	0	10.2		NA
Mildly ill (3)	19.3 (31)	10.7	38.8		
Moderately ill (4)	24.8 (40)	20.5	34.7		
Markedly ill (5)	39.8 (64)	50.9	14.3		
Severely ill (6)	13.0 (21)	17.9	2.0		
Extremely ill (7)	0 (0)	0	0		
**ADHD evaluation**					
DIVA 2.0 (A)	7.40 ± 1.25	7.53 ± 1.27	7.10 ± 1.14	2.008	0.046
DIVA 2.0 (H/I)	5.37 ± 2.82	5.68 ± 2.81	4.65 ± 2.75	2.146	0.033
CAARS (direct score)	21.94 ± 6.57	22.87 ± 5.85	19.82 ± 7.63	2.496	0.015
ADHD-RS	28.91 ± 10.32	31.46 ± 9.69	23.10 ± 9.40	5.077	<0.0001^*^
**Pharmacological treatment**					
Months in stable treatment	8.83 ± 12.65	4.97 ± 7.89	17.65 ± 16.60	5.103	<0.0001^**^

*NA: Chi-square test was considered not applicable when one or more of the cells had an expected count < 5*.

* *The result is statistically significant after Bonferroni correction*.

** *The result is statistically significant after logistic regression*.

### Logistic Regression Models

Finally, three logistic regression models were conducted using the variables that retained statistical significance after Bonferroni correction and considering insomnia disorder as the dependent variable (Table 5). The first model evaluated ADHD characteristics. It was observed that higher ADHD severity, ADHD-RS, and fewer months of stable ADHD treatment were independently related to a higher prevalence of insomnia disorder (*X*^2^ = 139.95; *p* ≤ 0.0001, Nagelkerke *R*^2^ = 0.571; constant value 6.239). The second logistic regression model analyzed medical and psychiatric comorbidities. In this model, current psychiatric disorders, previous history of anxiety disorders, and SUD were independently related to insomnia disorder (*X*^2^ = 47.84; *p* ≤ 0.0001, Nagelkerke *R*^2^ = 0.232; constant value 1.338). Finally, the relationship between psychological features and insomnia disorder was explored by using the variables that were statistically significant in the bivariate analysis. Low scores on physical and mental components of HRQoL were independently associated with a higher prevalence of insomnia disorder (*X*^2^ = 108.48; *p* ≤ 0.0001, Nagelkerke *R*^2^ = 0.467; constant value 7.358).

**Table 5:** Multivariate analysis of variables associated with insomnia disorder.

**Variables**	**Wald**	**gl**.	**Sig**.	**OR**	**95% CI**	
					**Lower**	**Upper**
**Model 1: ADHD-related variables**						
CGI	42.048	1	<0.0001	3.800	2.538	5.688
Stable ADHD treatment (months)	14.453	1	<0.0001	0.943	0.915	0.972
ADHD-RS	5.221	1	0.022	1.045	1.000	1.085
**Model 2: variables related to medical and psychiatric comorbidities**						
Any current psychiatric comorbidity	19.437	1	<0.0001	3.542	2.019	6.215
Previous anxiety disorder	7.441	1	0.006	2.263	1.258	4.069
Previous SUD	5.789	1	0.016	2.225	1.160	4.267
**Model 3: variables related to the psychological evaluation of comorbidity**						
Physical component of HRQoL	3.902	1	0.048	0.961	0.923	1.000
Mental component of HRQoL	58.064	1	<0.0001	0.874	0.844	0.905

## Discussion

In line with our hypothesis, the current study highlights that ADHD and insomnia disorder comorbidity in adults is highly prevalent. Moreover, patients who suffer both conditions are more clinically severe. Therefore, our hypothesis is confirmed as patients are characterized by a greater severity of ADHD, more psychiatric comorbidities, an unstable ADHD treatment, and a poorer health-related quality of life.

In the current sample, 44.4% of patients met the criteria for insomnia disorder according to DSM-5 and 63.9% had insomnia symptoms. This high prevalence of insomnia disorder and insomnia symptoms in ADHD patients is in line with the prevalence of insomnia symptoms previously reported to be between 43 and 80% (11, 12, 14). These results may contribute to delimiting this wide range and provide more insights into the prevalence of insomnia symptoms (better known) and insomnia disorder (scarcely reported) in adults with ADHD.

Current results on ADHD presentations and insomnia disorder were similar to previous studies that describe combined presentation is more associated with insomnia symptoms (11). However, it is important to highlight that some studies reported worse sleep quality and greater fatigue for the inattentive presentation (13), while other investigations found no differences among ADHD presentations (12). In any case, differences among ADHD presentations may be related or mediated by ADHD severity (50). Our results point out that ADHD severity is independently associated with insomnia disorder. These findings reflect the negative impact that both hyperactivity/impulsivity and attentional symptoms severity have on sleep onset and maintenance (9, 51).

When analyzing sociodemographic variables, only unemployment was significantly associated with more risk of insomnia disorder. These results have been already reported in previous studies, suggesting that unemployment is associated with poorer mental health and lower physical activity, which could cause insomnia (52). Note that other sociodemographic characteristics (e.g., sex, age, civil status) were not significantly related to insomnia disorder.

In line with other studies, patients with insomnia disorder had higher BMI (53). Obesity is usually related to greater morbidity and, in young adults, also to a worse lifestyle and health factors that may bring on the presence of insomnia disorder. Regarding medical conditions, due to the sample size, the current study was not able to find the results from previous reports about how insomnia disorder is related to a higher prevalence of hypertension, diabetes, gastrointestinal diseases, and cardiologic problems (54, 55). Only a higher frequency of pain syndrome was observed among patients with insomnia disorder, as well as their respective analgesic treatment.

Congruently with prior research (9, 56), psychiatric comorbidities were independently related to insomnia disorder. Regarding specific comorbidities, previous history of anxiety disorders and SUD, as well as current anxiety disorders, mood disorders, and SUD, were statistically significant in the bivariate analysis. SUD patients use to have a higher prevalence of insomnia disorder due to the disruptive effect that drugs of abuse and alcohol have on sleep (57). This interferes with the ease of falling asleep, increasing the difficulty in maintaining sleep and altering the cycling of the sleep stage. The relationship of previous SUD with insomnia disorder could be explained by the sleep disruptions that drug consumption may have generated, as well as the lack of healthy habits usually associated. Additionally, personality disorder comorbidity was statistically significant, specifically those from cluster B. Interestingly, some highly characteristic traits of these disorders such as impulsive behavior and emotional instability have been linked to a higher prevalence of insomnia and sleep disturbances (58). Additionally, regarding psychopharmacological treatments used for psychiatric comorbidities, our data showed a low frequency of prescription, which could be related to the high prevalence of psychiatric comorbidity.

Regarding pharmacological treatment, stimulant medications have shown to have a greater effect size on ADHD symptoms compared with atomoxetine (59). Probably, the drug dose and formulation are among the main factors that influence insomnia as an adverse effect. While some medication action can exceed 12 h, others last between 4 and 8 h. Although this adverse effect seems to be mitigated after 2 months of treatment (26, 27), it is possible that the long release presentations may cause greater insomnia by extending their effect until the beginning of the night.

Our results are congruent with the studies that indicate that pharmacological treatment would improve insomnia symptoms (11), even in adult drug-naive ADHD patients (60). Subjects who were in pharmacological treatment showed significantly less prevalence of insomnia disorder, specifically those who were prescribed with methylphenidate. Note that longer periods of stable treatment were associated with lower rates of insomnia disorder (even when confounding variables were controlled for). Some authors describe that the pharmacological treatment effect on insomnia symptoms should be analyzed from a broad perspective that considers the clinical characteristics and severity of ADHD, suggesting the existence of a specific phenotype in ADHD patients who might respond better to this treatment (61). In any case, it is important that more research is conducted on this issue.

Concerning insomnia phenotypes, when considering the different risk factors, only age had statistically significant association. As previous studies reported (62, 63), younger patients had more frequently sleep-onset insomnia phenotype. However, in contrast to those studies, no differences were found regarding psychiatric and medical characteristics (63). This finding could be related to a sample bias as the current sample had a high prevalence of psychiatric comorbidities.

The current analysis exposed a higher incidence of insomnia disorder in patients who use screens in the half-hour before bedtime. Exposure to blue light from these devices has been associated with a greater alteration of circadian rhythms, as well as a higher prevalence of sleep-onset insomnia and poorer sleep quality (64). Some authors point out an excessive use of technology at night in the ADHD population (65).

Regarding health-reported quality of live, insomnia disorder was independently related to a negative impact on both physical and mental components of HRQoL. Only patients without insomnia disorder presented normal scores in the physical component of HRQoL. Physical and mental components were affected in patients with insomnia disorder compared with normative data (66). Therefore, insomnia disorder is associated with functionality and worse HRQoL in these patients. On the other hand, different studies have found a negative correlation between ADHD symptoms, the severity of ADHD, and its comorbid pathologies, and the scores on the quality of life scales (67–69). Our results point out that insomnia disorder contributes to worsening of this perception in adults with ADHD, and conversely, its treatment and remission may imply an improvement in the quality of life.

Finally, when characterizing the insomnia symptoms group, our results point that patients with insomnia symptoms had lower clinical severity of ADHD according to the ADHD-RS and a greater number of months of stability of the ADHD treatment. Furthermore, our results showed lower psychiatric comorbidity and better mental component of the HRQoL in patients with insomnia symptoms when they were compared with patients with insomnia disorder. These results, which support our hypothesis, could contribute to identify the factors of severity of ADHD, psychiatric comorbidity, and quality of life that could be associated with the transition between both clinical categories: insomnia disorder and insomnia symptoms.

This study should be analyzed regarding its limitations. First, objective or electrophysiological measures of sleep such as polysomnography or actigraphy were not used. The use of these instruments would provide more strength to the results. Second, the cross-sectional design does not allow analyzing the bilateral association. Third, the effect of pharmacological prescriptions for the treatment of psychiatric comorbidities, specifically mood disorders, was not controlled. Fourth, except for the use of screens before sleeping, no other patient sleep hygiene measures were recorded and neither do the patients diet characteristics. On the other hand, this research has important strengths. To the best of our knowledge, this would be the first published study evaluating the relationship of adult ADHD with insomnia disorder according to the diagnostic criteria of DSM-5 in adult patients. Furthermore, to avoid confounding factors, those patients who suffered any other sleep disorder that could be confused with insomnia disorder (e.g., obstructive sleep apnea, circadian rhythm disorder) were excluded from the study. Finally, we must emphasize that the sample has included naive patients and treated patients, which allows us to better assess the impact of the treatment, as well as to bring the results closer to the reality of clinical practice.

The current study points out that adult ADHD patients with insomnia disorder have a worse clinical presentation due to a higher ADHD severity, more comorbidities, and worse quality of life. These results may contribute to a better understanding and clinical approach of insomnia disorder in the context of a wide and heterogeneous pathology such as ADHD, by allowing to identify some of the sociodemographic, clinical, and comorbidity characteristics associated with both disorders when they occur together.

## Data Availability Statement

The datasets generated for this study can be available on request to the corresponding author. Part of the data may not be allowed for distribution to others than the research group that conducted the study as may violate ethical and/or legal regulations of written consent.

## Ethics Statement

The studies involving human participants were reviewed and approved by Clinical Research Ethics Committee of the Hospital Universitari Vall d'Hebron. The patients/participants provided their written informed consent to participate in this study.

## Author Contributions

CF, CD, LG-L, and JR-Q conceived, designed, and supervised the study. CF, CD, VR, and MC contributed data or analysis tools. CF, VR, and MC collected the data. CF, CD, LG-L, and RP-A performed the data analysis. CF, CD, VR, LG-L, RP-A, MC, and JR-Q wrote the manuscript. JR-Q secured funding for the study. All authors contributed to the article and approved the submitted version.

## Conflict of Interest

CF has received fees to give talks for Shire/Takeda and Rubio. VR has received travel awards (train tickets + hotel) for taking part in psychiatric meetings from Shire in the last 3 years. LG-L has received fees to give talks for Janssen-Cilag, Lundbeck, Servier, Otsuka, and Pfizer. RP-A has received fees to give talks for Exeltis, Lundbeck, MSD, Mundipharma, and Takeda. JR-Q was on the speakers' bureau and/or acted as consultant for Eli-Lilly, Janssen-Cilag, Novartis, Shire, Takeda, Bial, Shionogui, Lundbeck, Almirall, Braingaze, Sincrolab, Medice, and Rubió in the last 5 years. He also received travel awards (air tickets + hotel) for taking part in psychiatric meetings from Janssen-Cilag, Rubió, Shire, Takeda, Shionogui, Bial, Medice, and Eli- Lilly. The Department of Psychiatry chaired by him received unrestricted educational and research support from the following companies in the last 5 years: Eli-Lilly, Lundbeck, Janssen- Cilag, Actelion, Shire, Ferrer, Oryzon, Roche, Psious, and Rubió. The remaining authors declare that the research was conducted in the absence of any commercial or financial relationships that could be construed as a potential conflict of interest.
